# Corrigendum to ‘Reliability and validity of the multidimensional dyspnea profile in hospitalized Chinese patients with respiratory diseases’

**DOI:** 10.1177/20503121211066430

**Published:** 2021-12-23

Huaying Chen, Yamin Li, Weihong Wang, Huilin Zhang, Na Nie, Jinnan Ou and Lezhi Li. Reliability and validity of the multidimensional dyspnea profile in hospitalized Chinese patients with respiratory diseases. *SAGE Open Medicine*. Volume 9: 1–9. doi: 10.1177/2050312120965336

An error was introduced in the first name of the corresponding author. The correct name of the author reads as follows: Lezhi Li

